# Patients’ perspectives of the effects of a group-based therapeutic patient education program for bipolar disorder: a qualitative analysis

**DOI:** 10.1186/s12888-022-04241-2

**Published:** 2022-09-23

**Authors:** Mélanie Duval, Yves-Antoine Harscoët, Julien Jupille, Marie Grall-Bronnec, Leïla Moret, Marion Chirio-Espitalier

**Affiliations:** 1 Public Health Department, University Hospital of Nantes, 44093 Nantes, France; 2 Psychiatry and Mental Health Department, University Hospital of Nantes, Nantes University, 44000 Nantes, France; 3 SPHERE (MethodS in Patients Centered Outcomes and HEalth ResEarch), Nantes University, Tours University, University Hospital of Nantes, INSERM,, 44000 Nantes, France

**Keywords:** Bipolar disorder, Personal recovery, Therapeutic education, Psychoeducation, Qualitative research, Patient experiences

## Abstract

**Background:**

Few qualitative studies have explored the impact of group-based psychoeducation programs from the perspective of patients with bipolar disorder, and no studies to date have examined the effects of such programs on patients’ personal recovery. The aim of this study was to explore the effects of a group therapeutic education program on the personal recovery of people with bipolar disorder and its determinants.

**Methods:**

Three professionals conducted semistructured interviews with 16 patients who participated in 9 weekly sessions of four separate bipolar therapeutic education programs. The interviews were transcribed verbatim and analyzed inductively by two of the professionals using the thematic analysis method.

**Results:**

Three main themes emerged from the interviews: the elements of therapeutic education, the experience of therapeutic education and the changes facilitated by therapeutic education. The changes reported by the participants included the evolution of the patient’s relationship with the disorder, improvement in the patient’s knowledge of the disorder, improvement in disorder management throughout daily life in general, and development of psycho-social skills and social relationships.

**Conclusions:**

This study provides support for the beneficial impact of group therapeutic education programs on the personal recovery of people with bipolar disorder. These programs improve all dimensions of recovery according to the CHIME model, with connectedness, hope and empowerment being the main dimensions impacted. Our results indicate that therapeutic group education programs can be beneficial for people with bipolar disorder at any point during their experience of the disorder, with the potential exception of periods of thymic decompensation.

**Supplementary Information:**

The online version contains supplementary material available at 10.1186/s12888-022-04241-2.

## Introduction

According to the World Health Organization (WHO), bipolar disorder is one of the ten most disabling chronic disorders [1]. While its prevalence worldwide is 2.6% among the general population [2], the prevalence of this disorder in France has been estimated to be between 1% and 2.5% [3]. This pathology is marked by a high rate of suicide, between 11 and 19% [1], and changes in social and professional functioning, which persist beyond episodes of thymic decompensation [4].

To remedy these consequences with respect to the daily life of patients with bipolar disorder, complementary approaches to drug treatment and psychotherapy have been developed over the past twenty years, i.e., group-based psychoeducation interventions. The benefits of these approaches on the course of bipolar illness have been widely explored by quantitative studies: the use of psychoeducation reduces the rate of relapse [5–8], increases the delay between two episodes of decompensation [5, 7, 9], decreases the number and duration of hospitalizations [5–7] and improves the adherence to treatment [5–8] and social functioning of patients [5, 10].

However, few qualitative studies have explored the impact of group psychoeducation programs from the patients' perspective. A few qualitative studies assessing the acceptability, feasibility and effects of outpatient or inpatient psychoeducation programs have found evidence of increased acceptance of the disorder, improved self-confidence and health behaviors, expanded social networks, increased treatment literacy and an improved therapeutic alliance with caregivers [11–13].

Furthermore, no qualitative studies have investigated the effects of psychoeducational approaches on the personal recovery of patients with bipolar disorder. The notion of personal recovery refers to the ability to live a satisfying and fulfilling life, regardless of the course of the illness. Such personal recovery represents a way out of mental illness that differs from ordinary recovery. Indeed, the aim of personal recovery is not the disappearance of the symptoms but an improvement of the person's future. The suppression of symptoms is no longer the priority for the person and the associated care providers; the emphasis is rather on the personal wishes of the individuals in question, their strengths and the means necessary to achieve their desires [14, 15]. This notion of recovery originates from the users of psychiatric care themselves and not from their care providers. The development of this notion has been supported by the results of studies that have investigated people with psychiatric illnesses over the long term, which have highlighted the possibility of regaining a satisfactory social, personal and professional life, despite the persistence of the symptoms of the illness. Personal recovery is a deeply personal process, and the path to such recovery is not linear. Psycho-educational approaches could be an interesting tool to assist patients on the road to recovery, as such approaches focus on the management of the patients’ daily lives.

Several conceptual frameworks for personal recovery in the context of mental health have been developed over the past decade, which synthesize the experiences of personal recovery described by patients [16–18]. The best-known and most widely used model is the CHIME model, an acronym referring to connectedness, hope, identity, meaning in life and empowerment [19]. This model includes 5 dimensions corresponding to the 5 main known determinants of personal recovery. In our study, we sought to relate our findings to the dimensions highlighted by the CHIME model.

In our study, patients with bipolar disorder attended group therapeutic education programs. These programs are comparable to psychoeducation programs, but they differ slightly in that they are more personalized: the programs must be adapted to the needs of each participant. The aim of these programs is to provide the patient with the knowledge necessary to understand the disorder, its evolution and its treatment and to increase the patient’s skills in self-care and adaptation to allow the patient to become a real actor with respect to his own care [20].

The objective of our study was to explore the effects of a group psychoeducational approach, specifically a therapeutic education program, on the personal recovery of people with bipolar disorder and its determinants. We sought to understand the factors of the therapeutic education program that contribute to the patients’ recovery progress. As such, we also aimed to compare our findings with the dimensions included in the CHIME model.

## Method

### Intervention

The therapeutic patient education (TPE) program on which our study is based is administered at the CReSERC (Centre Référent en Soins d’Education thérapeutique et de Remédiation Cognitive), a service associated with the Nantes University Hospital. This TPE is a specific program for patients with bipolar disorder and was created with the support of professionals from the Nantes University Hospital who were experts in TPE and who were evaluated every 4 years by the regional health agency (the most recent evaluation occurred in 2020). The TPE program follows the recommendations of the High Authority on Health, particularly in terms of involving coconstruction between users and carers [21]. The program includes 9 weekly group sessions lasting two hours each, which are led by a doctor/nurse pair, each of whom is trained in TPE. A peer helper also co-facilitates all sessions. This peer helper participates in the discussions and shares his experiences regularly when the situation allows. He shares his experiences concerning the disorder and his keys to recovery. Each group consists of a maximum of 10 participants, with the composition of the group remaining unchanged throughout the TPE cycle. Prior to participation in the TPE program, an individual educational diagnostic session is conducted. This session aims to determine the perceptions of the participant concerning his pathology and expectations of the program. The sessions address different topics, such as the various types of bipolar disorder, depression and manic/hypomanic episodes, treatments, and daily management of bipolar disorder. A session including relatives is also planned. The full content of the sessions is detailed in the appendix (Additional file 1). The order and content of the sessions can be adapted to suit the wishes and needs of the participants, which are defined during the first session. Practical tools that can be applied outside the hospital setting are provided to participants during each session. An individual post-TPE evaluation session is offered systematically to each participant within 4 months of the end of the program to discuss their experiences thereof.

### Design and recruitment

This paper presents a monocentric (i.e., patients were recruited from a single center, namely, the Nantes University Hospital in France), qualitative (i.e., no quantitative data were collected) study exploring patients’ perspectives, which was conducted by the CRESERC. Participation in the study consisted of a qualitative individual interview within 4 months of the final TPE session. During the individual educational diagnosis session, such participation was proposed to patients who were enrolled in two separate bipolar TPE programs that occurred simultaneously from 11 September 2020 to 11 November 2020. All patients were systematically invited to participate in the study, irrespective of whether they attended all the sessions. Following scrutiny of these participants’ interviews, to ensure data saturation, it was decided to include four additional participants from two other bipolar TPE programs lasting from 28 January 2021 to 2 April 2021. The reason for this addition was that new themes emerged during the analysis of the final initial interviews, which indicated that data saturation could not have been achieved using solely the initial interviews.

Patients were included if they met the following criteria: diagnosis of bipolar disorder type 1 or 2 made by a psychiatrist and confirmed at the Mini International Neuropsychiatric Interview (MINI), age between 18 and 70 years, and current medical management of the bipolar disorder and treatment with a mood stabilizer. Subsequently, patients who were minors, who had poor French language skills or obvious cognitive impairment as measured by the Montreal Cognitive Assessment (MoCA), or who were under guardianship were excluded. Patients were informed of the study via an information letter, and their written non-opposition was provided.

### Data collection

Patients’ perspectives were collected during semistructured interviews conducted by three professionals with different profiles: an advanced practice nurse who regularly conducts TPE sessions (7 interviews), a medical intern who was trained in TPE (5 interviews) and a sociologist who was not trained in TPE (4 interviews). The participants had no prior relationships with the interviewers. The three researchers followed an interview guide containing open-ended questions, which was created in collaboration with a medical doctor who was trained in qualitative analysis (Additional file 2). The questions were developed following a literature review that identified key themes related to the research question. The initial questions asked about the patient’s experience of bipolar disorder in general and contextualized the contributions to and experiences of TPE among participants. Subsequently, four major areas were addressed: expectations and motivations regarding participation in the TPE program, effects related to the program, evaluation of the TPE process and suggestions for improvement. The interviews were conducted in a flexible manner to allow participants to express themselves freely and to highlight all themes that corresponded to the objectives of the study. The feasibility and relevance of the interview guide was tested prior to the study by reference to two patients who had participated in a previous TPE program and who agreed to participate in the interview. The interviews, which were initially planned to be conducted face-to-face, were conducted by telephone or videoconference due to the contact precautions in place during the COVID-19 pandemic. These interviews were conducted between 1 and 3 months after the participant’s final TPE session. These interviews were distinct from the individual post-TPE evaluation sessions, which were an integral part of the TPE cycle. Each interview lasted between 30 min and 1 h and 15 min. All interviews were recorded and transcribed with the patient's consent.

In addition, to describe our study sample in brief, sociodemographic data were collected at the time of inclusion: age, employment, family status, year of bipolar disorder diagnosis, year of most recent hospitalization and type of bipolar disorder.

### Analysis

All interviews were transcribed manually. Two of the authors, the medical intern and the sociologist, analyzed these interviews qualitatively using the thematic analysis method. This pragmatic approach seemed to us to be the most suitable for the objective and design of our study. This approach consists of identifying themes from the quotations of the participants, organizing these themes into main themes and subsequently establishing links among these main themes.

As described by Braune and Clarke in 2006 [22] and explained further by Paillé and Mucchielli in 2008 [23], this method consists of 6 steps:

- Gaining familiarity with the data
- Generating initial codes
- Searching for themes
- Reviewing themes
- Defining and naming themes

- Producing the report

Before starting the actual analysis, the researchers read all the interviews to obtain an overview of the patients’ perspectives. Subsequently, they identified the themes emerging from each interview in relation to the research question inductively, without any preconceived ideas, i.e., without any preestablished thematic grid. The resulting themes reflected not only the questions asked during the interviews but also subjects not mentioned in the interview framework. This expansion was possible because the interviews were conducted in a flexible manner, affording patients the opportunity to address unexpected topics. The thematic grid was created after the analysis of the initial interviews and was modified as subsequent interviews were read.

The interviews were analyzed separately by the two researchers who were blinded to each other. Consultation between these two researchers and a psychiatrist took place following the coding of the interviews to compare the themes identified by each researcher and to establish links among the main themes. All the themes were identified by the two researchers, and consensus among the members of the research team concerning the organization of the main themes was reached. Data saturation, which can be defined as a situation in which no new themes are identified in the analysis of the final interviews [24], was reached. No new themes related to the research question appeared in the coding of the final three interviews.

The interviews were coded with the help of qualitative analysis software, i.e. NVivo version 12.

### Ethical considerations

This study was approved by the Nantes Health Ethics Group (GNEDS). An informational note concerning the study, including the procedures for the collection and recording of oral data, was provided to participants, and their oral consent was received. Participants were also informed that they could freely withdraw from the study at any time.

## Results

### Sample characteristics

Sixteen patients were included in the study; most patients were women, the mean age was 37 and the mean duration of disorder was 16 years. All characteristics of these participants are described in Table 1. The demographics of our sample are similar to those of groups that are usually encountered in TPE programs.

**Table 1:** Sociodemographic characteristics of participants

Patients’ characteristics (*N* = 16)	
**Gender**	
Male	4
Female	12
**Age in years**	
Range	20–51
Mean	37
**Family status**	
Single without children	5
Single with children	2
Part of a couple without children	4
Part of a couple with children	5
**Employment status**	
Student	2
Employed	6
Unemployed	8
**Time since onset of symptoms in years**	
Range	5–27
Mean	16
**Time since diagnosis in years**	
Range	1–16
Mean	5
**Type of bipolar disorder**	
Type 1	5
Type 2	8
Undetermined	3

Three main themes emerged from the qualitative analysis of the interviews: the elements of therapeutic education, the experience of therapeutic education and the changes facilitated by therapeutic education. The themes and subthemes identified in association with each main theme are listed in Table 2.

**Table 2:** Main themes, themes and subthemes arising from the qualitative interviews with participants

Themes arising from the qualitative interviews
***Main theme 1*: Elements of therapeutic education**
**Theme 1: An atmosphere of trust and security**
Rules for group life
Empathy
Possibility of going outside during the sessions
Freedom of speech and opinions
Horizontality of the patient-caregiver relationship
**Theme 2: Interpersonal factors**
Group heterogeneity
Peer support
**Theme 3: Session facilitators**
**Health staff**
Complementary skills
No hierarchy among caregivers
Empathy and benevolence
**Peer helpers**
Sharing experiences and solutions
Moral support for participants
Sources of hope
Links between caregivers and participants
**Theme 4: Structure of the sessions**
Participatory sessions
Varied animation
Planning of sessions in accordance with participants’ wishes
***Main theme 2*: Experience of therapeutic education sessions**
**Theme 1: Cohesion and group spirit**
Good atmosphere
General positive experience of the sessions
Disappointment related to the loss of group cohesion when sessions ended
**Theme 2: Emotional difficulties**
Confronting the reality of the disorder
Confronting the suffering of other participants
Resurgence of painful memories
**Theme 3: Time management**
Lack of time
***Main theme 3*: Changes facilitated by therapeutic education**
**Theme 1: Evolution of the patient’s relationship to the disorder**
Awareness of the disorder
Confirmation of the diagnosis of bipolar disorder
Improved acceptance of the disorder
Relegation of the disorder to the background
Hope for recovery
**Theme 2: Improving knowledge of the disorder**
Better understanding of the disorder
Reinforcing prior knowledge
Correcting false information
**Theme 3: Improving disorder management**
Acquisition of tools
Implementation of new strategies
Validation of preexisting strategies
Improved communication concerning the disorder
Coconstruction of the treatment with the psychiatrist
**Theme 4: Development of psychosocial skills**
Removing guilt
Increasing in self-confidence
Self-affirmation
**Theme 5: Development of social links**
Feeling of belonging to a group
Reduction in the feeling of loneliness resulting from the disorder
Expansion of social networks
Identification and selection of resource persons
Development of peer support
**Theme 6: Change in the management of daily life**
Increased peace of mind and serenity
Taking a step back from everyday situations
Increased freedom to act

### Elements of therapeutic education

This main theme relates to factual elements discussed during the TPE sessions that participants identified as beneficial. Four main elements were identified by participants.

#### Theme 1: An atmosphere of trust and security

Participants spoke positively about the rules for group living that were established during the first TPE session, which were suggested by the facilitators and approved by all the participants. These rules focused on listening to each other, nonjudgment and benevolence. As a result, participants experienced a great deal of empathy in the discussions.

“The atmosphere was pretty good, people listened to each other, we tried to be empathetic… without judgment. There were rules that were put in place from the beginning. We listened to each other.” (P2, female, 42 yrs).

Some participants felt reassured by the possibility of leaving the room freely and being accompanied in case of difficulties.

“They explained to us that if there was an emotional outburst, we could leave the room and, if we wished, be accompanied by a caregiver to be able to express ourselves because we had a more important emotion than usual that overflowed… That was very clear from the beginning.” (P4, male, 40 yrs).

Many participants noted that they had a great deal of freedom to speak and that everyone's opinion was considered and respected.

“Everyone could express themselves, for those who wanted to express themselves. I thought it was well framed; we could talk about any type of subject.” (P11, male, 39 yrs).

Most participants perceived a horizontal relationship between themselves and the facilitators. Caregivers were not placed in a position of superiority but shared their knowledge with the participants in a manner that established that knowledge as being of equal value.

“Compared to other forms of care, I would say that it introduces horizontality into the relationship, a bit like peer support. It’s not the psychiatrist, the expert, who sees you for two minutes during the day and gives you information from his expert position. Here, there are a lot of discussions that are conducted horizontally between us, and that change everything.” (P8, female, 43 yrs).

All of these components allowed the participants to feel confident when expressing themselves and discussing personal issues.

“There was a lot of care. I think we all felt really safe, which meant that over time we started to confide in each other a lot more, to talk about our personal lives a lot more, whereas at the beginning I was really very closed.” (P15, female, 42 yrs).

#### Theme 2: Interpersonal factors

The heterogeneity among the group members was noted as a positive aspect by many participants. The presence of participants of different ages allowed the young people in the group to imagine their future with the disorder. In addition, some participants were able to become aware of the positive aspects of their lives and the support of those around them by communicating with other participants from more disadvantaged social backgrounds.

“The fact that we are of different ages provides a lot, I think, because we have different visions. We can learn a lot from older people, whereas if we had been a group of young people, it would have been more complicated because we would all have been at the same stage. So it really allows you to see a very different panel and to learn from everyone.” (P6, female, 20 yrs).

Moreover, the groups were composed of people with bipolar disorders of different levels of severity. For more than half of the participants, this heterogeneity in terms of the severity of the disorder enabled the participants who were least affected by the disorder to relativize the importance of their disorder and allowed the participants who were most affected to imagine future improvement.

“When you hear people talking, you think “wow”, some of them have really experienced terrible things. And, uh, it’s a little uncomfortable at first, and then sometimes, I said to myself, I'm not as affected as that.” (P10, female, 39 yrs).

The sharing of experiences with peers was perceived to be a benefit of the TPE sessions by all participants. Participants were able to provide solutions to the problems faced by others. This support added value to the solutions proposed by the caregivers because it was based on the participants' own experiences.

“All I learned was … it was each other’s testimonies. Yes … but it became even more obvious to me. Yes, I had read it in books. Now it's even more concrete.” (P3, female, 43 yrs).

This situation also made it possible for the patients to value the experiential knowledge of all participants, which complemented the theoretical knowledge provided by caregivers or other sources.

“Oh yes, for me, it’s important to be able to help people in my situation, to give advice, to share something that could be similar… a war. I like it very much. I didn’t know this notion of peer support before. I really like this concept because it's really a help, it’s something we know about.” (P10, female, 39 yrs).

#### Theme 3: Role and posture of session facilitators

##### ***Health staff***

Half of the participants highlighted the complementarity among the different caregivers who facilitated the sessions.

“I find that having several caregivers already helps them to cover more subjects. So, everyone has their own experience and skills; that's a good thing.” (P4, male, 40 yrs).

The participants appreciated the absence of any hierarchy among the caregivers, which was helpful in promoting the horizontalization of the relationship between caregivers and participants. The caring and empathetic attitudes of the caregivers were also unanimously praised.

“The care providers, we’ll say that they were all on the same line. And it wasn’t ‘I’m a psychiatrist, you’re a nurse’. You could see that it was quite uniform. Yes, I think I would have felt very uncomfortable if there had been firm limits within the team. Since nothing was brought by the care providers except benevolence and then a human uniformity, it clearly helped me to feel at ease.” (P7, male, 33 yrs).

##### ***Peer helpers***

The presence of a peer helper during the TPE sessions was noticed and considered to be a real asset by the majority of participants. The peer helper guided the participants by sharing her experience of the disorder and strategies for living with it and provided moral support to people facing difficulty during the sessions.

“She has answers that we didn’t have before. So, she guides us, she’s always positive, she pushes us up, I mean, I found her to be a source of solutions. She’s really very positive, um, unfailingly positive.” (P9, female, 42 yrs).

The presence of the peer helper was a source of hope for almost half of the participants. She gave the participants a glimpse of a positive progression of life with bipolar disorder.

“Yes, it shows that we’re going to make it. That life will be what it is with this bipolar disorder, but we’re going to make it.” (P12, male, 40 yrs).

Some participants found that the peer helper occupied an intermediate position, thus bridging the gap between the caregivers and the participants.

“I think it provides a link between the group and the caregivers, actually. Maybe because they have a longer recovery. The fact that they’ve been in recovery longer, maybe they’ve been able to digest some of the emotions and concepts that we have trouble verbalizing. The peer helper allows for a transfer of medical knowledge between peer helper and patient. And it also allows, I think, for a reduction in this hierarchy between patients and doctors.” (P10, female, 39 yrs).

#### Theme 4: Structure of the sessions

Almost half of the participants mentioned the participatory nature of the TPE sessions. The frequency of the group work allowed participants to become more involved in their care and to find solutions to problems on their own.

“Within the group itself, there were a lot of discussions; we talked well, we worked well together. It’s good because we’re actually looking for the solution by ourselves.” (P9, female, 42 yrs).

Some participants noted that the sessions exhibited substantial variation and used appropriate educational tools.

“I thought it was really well done and that we used different tools, which could vary from session to session… sometimes we were in a group, sometimes we were in pairs… sometimes there was a slide show, sometimes it was just someone talking… I thought it was really varied in the way that the sessions were facilitated and that it adapted well to each session.” (P5, female, 25 yrs).

Several participants also appreciated the freedom they were given to plan the sessions and choose the topics that would be covered throughout the TPE cycle.

“I really liked the fact that we chose the topics we wanted to talk about. I didn’t expect that. I thought it would be a predefined program. The fact that you can vote for the topics, I think it’s really good because it’s more personalized. It’s more focused on our personal needs.” (P6, female, 20 yrs).

### Experience of therapeutic education sessions

This main theme describes the feelings and emotions experienced by the participants during the TPE sessions. Three themes were found during the interviews.

#### Theme 1: Cohesion and group spirit

All the participants felt a strong sense of group cohesion, despite their diversity of opinions and characters.

“I really felt the humanity because I found myself in a really nice group, with very endearing people in whom I also recognized myself.” (P15, female, 42 yrs).

The special atmosphere that developed during the TPE sessions contributed to a positive experience of TPE for almost all participants.

“I felt good during the sessions. We were very welcome… We felt good, we didn’t want to leave.” (P3, female, 43 yrs).

Some participants expressed difficulty due to the fact that the sessions ended abruptly, while others regretted the fact that this group cohesion did not continue after the TPE sessions.

“I thought it was sad that none of the patients wanted to make the effort to go for coffee afterward or give a phone number. I wasn’t very comfortable taking someone’s phone number… I guess the other patients weren’t either. I was a little disappointed.” (P7, male, 33 yrs).

#### Theme 2: Emotional difficulties

These sessions were emotionally charged for most participants.

First, participants were confronted with the reality of their disorder, even though some were initially in denial. The sharing of each other's problems may have caused some participants to become temporarily discouraged in anticipation of the difficulties that would emerge.

“In the group, there were some who had been diagnosed very young like me and who, at 60, had gone through a lot of phases. So, it’s complicated to see that anyway. It’s not that I was disappointed, but I imagined that there were people who had succeeded… I told myself that it was easier for the others, but in fact, no.” (P14, female, 30 yrs).

Second, almost half of the participants reported feeling the pain expressed by other group members.

“It’s true that sometimes, when hearing the testimonies of others, things were quite violent. These are things that I don’t experience. So, at times, I told myself that I had a lot of empathy.” (P1, female, 32 yrs).

Finally, the sharing of experiences led to the emergence of painful memories related to the disorder for some participants.

“Obviously, sometimes, certain sessions or certain topics of discussion stir things up inside, and so it really got me thinking… Well, it’s not always pleasant.” (P15, female, 42 yrs).

#### Theme 3: Time management

Half of the participants felt that there was insufficient time to discuss all the topics they wanted to address. This feeling was most prevalent among participants who had high expectations regarding TPE.

“I thought that it was too short, actually. Many of us found it too short. So, there were some topics that were not covered enough, such as treatments. I think there were a lot of people who needed to talk about their treatments. And so, two hours, it was a bit short for everyone to talk, since at each meeting we were completely overflowing.” (P10, female, 39 yrs).

### Changes facilitated by therapeutic education

This main theme pertains to the changes caused by the TPE program. Six main changes, both related to the disorder and to daily life in general, were reported by the participants.

Most patients had high expectations regarding their participation in TPE and were highly motivated. Their main expectations were to encounter the testimonies of other people with bipolar disorder, to obtain more knowledge regarding the disorder and to evaluate themselves in comparison with other people with bipolar disorder.

A few participants explained that not all their expectations had been met by the TPE sessions. The reasons given for this failure were a lack of time to address all the desired topics in depth, frustration that the support would eventually come to an abrupt end and disappointment regarding their inability to maintain relationships with other members between sessions.

#### Theme 1: Evolution of the patient’s relationship to the disorder

The majority of the participants’ relationships to the disorder changed in different ways throughout the TPE sessions.

A third of the participants explained that their participation in the TPE had led to increased awareness of the disorder, which had previously seemed vague and distant.

“I’ve become aware of some silly things, that I’m ill… Yes, to hear myself say that, it is complicated. It’s not really integrated into my daily life yet, well, into my everyday life. With my relatives, we don’t really talk about it, so it’s difficult to pretend that I don’t have my weaknesses. But a step has been taken, and I know there are things I can’t do anymore, and I became aware of this in the TPE program.” (P1, female, 32 yrs).

For some participants who were in denial regarding their illness, TPE even removed their doubts concerning their diagnosis of bipolar disorder.

“I didn’t feel sick, after all. I said to myself, ‘No, that’s a myth’. And in fact, meeting people with this same pathology and talking to them, I found myself in them. What they were saying, I was experiencing it too.” (P10, female, 39 yrs).

In addition, approximately one-quarter of the participants stated that they became more accepting of their illness and its consequences due to the TPE sessions.

“Yes, more an acceptance of all that. I accept it better, and I accept my illness better anyway.” (P4, male, 40 yrs).

For one-third of the participants, the TPE also helped them to put their disorder into the background and to envision the possibility of new life projects despite their disorder. They explained that they abandoned their previous identity as nothing more than a sick person.

“So, it gave me even more courage, a new breath to continue in my approach, to put myself forward, to stop hiding behind my illness, to really be me, to feel much more alive in my life, because it's true that I was hiding a lot for fear that I would experience a crisis or that something would happen. Of course, my illness is there, but I don’t want to put it forward anymore; I really want to put it in a box”. (P16, female, 36 yrs).

Finally, half of the participants revealed that the TPE sessions gave them hope that they could improve their well-being quality of life as well as, more generally, hope for recovery. TPE was an important source of motivation with respect to the management of their psychological health.

“There is this thing, yes, it is possible. It’s feasible, it’s not impossible, it’s within reach, we’ll say. You have to work, obviously, but it’s not impossible. And that’s the message I took away from it, that despite everything, you have to persevere.” (P6, female, 20 yrs).

“Well, it reinforces the idea that I can get out of it, and that I can’t heal, but I can restore stability in my life and when there are crises one way or the other, it allows me to say that I have the tools to detect them in advance and implement strategies to avoid things getting worse.” (P4, male, 40 yrs).

#### Theme 2: Improving knowledge of the disorder

All the participants reported that they improved their knowledge of bipolar disorder during the sessions.

This improvement enabled them to understand their illness and their own functioning more clearly.

“We saw what a pattern of vulnerability was, everything that could cause us to enter different phases. And it’s true that there are certain things that I couldn’t identify on my own before. I understood that—when I told you that I didn’t know how my first depressive phase started at the very beginning—well, it was when I stopped working part time in restaurants. And I didn’t think that could be an event that could trigger a crisis…” (P14, female, 30 yrs).

In addition, according to one-third of the participants, the TPE enabled them to confirm and reinforce their previous knowledge concerning the disorder. Discussing the disorder with caregivers and other group members made this knowledge more concrete.

“Yes, there’s the internet, there are books. There are things I knew, but it’s not the same to look at things on the internet or in a book as it is to meet people, to have contact, to hear things. It’s different, it doesn’t have the same impact, I think.” (P14, female, 30 yrs).

Some participants also had the opportunity to correct misinformation regarding bipolar disorder.

“Even if I didn’t learn a lot about the disea disorder se as such, it may have rectified some things… Some bad information I had…” (P6, female, 20 yrs).

#### Theme 3: Improving disorder management

During the TPE sessions, participants were introduced to tools to facilitate the assessment and management of the disorder. Most participants agreed with the effectiveness of these tools and integrated those that best suited them into their daily lives.

“I like to do the ‘mood temperature’. It allows me to try and manage my day. I do that after my meditation. Yes, because having tools to fight the symptoms, up or down… It’s like we’re actually armed against our disorder, and it’s there, but we can manage it.” (P9, female, 42 yrs).

As a result of the TPE, the majority of the participants reported implementing behaviors and strategies to manage their disorder throughout their daily lives. These changes included slowing down the pace of their lives to save energy or stay calm during an excited phase, taking more time for themselves, listening more to their own needs, and improving their lifestyles.

“There’s a term that came out on its own and that gave me a trigger. I said at one point that when you’re depressed, you tend to go into “energy conservation”. It allowed me to say to myself that ultimately, when I face difficulties in everyday life, I should not hesitate to take a short 15-min nap to be able to leave much less tired. And not to be afraid, not to tell myself that I'm lazy because I save my energy.” (P4, male, 40 yrs).

“I am careful not to go to bed too late. I try not to eat too late at lunchtime, not to skip meals… These are all very simple things, but I wasn’t aware of that, I didn’t see how it could make me sick.” (P1, female, 32 yrs).

In addition, TPE enabled half of the participants to validate preexisting strategies that they had instinctively adopted to cope with their fluctuating moods.

“It strengthened me in some ways; I thought I was right to listen to music, to read, to go out, to walk in nature… things that were positive.” (P3, female, 43 yrs).

Moreover, some participants reported better communication concerning their illness with their relatives as well as with their psychiatrists. These participants felt better “armed” to express themselves with respect to their pathology than they were prior to the sessions.

“It’s great to know how to defuse things with the people close to me… and even with my psychiatrist. I wouldn’t have believed it, but the fact that I know more about the illness makes it easier to talk about it, to talk about medication. There is better communication with him. The relationship is much more direct.” (P1, female, 32 yrs).

Finally, some participants reported an evolution in the way in which their psychiatric management was constructed. As a result of the TPE, they wanted to coconstruct their medical care with the psychiatrist and to discuss different therapeutic options actively.

“And then it created a debate with my psychiatrist. He had never talked to me about my bipolarity and all that. And the fact that I told him that I had been to TPE… ‘I’m more of a type 2…’ He totally agreed. He explained to me why he was putting this treatment there and not that one. Now I know something about it, and he sees that scientifically, there are things I know about, so he is interested.” (P1, female, 32 yrs).

#### Theme 4: Development of psychosocial skills

Half of the participants reported a reduction in their sense of guilt due to being ill, being helped with employment or receiving financial support. The participants explained that they exercised more benevolence toward themselves and finally allowed themselves to take advantage of the right to happiness.

“It made me feel less guilty. I was really guilty… For example, I went on a journey, I’m going to take advantage of it, I’ve got this chance. I can leave, taking care of myself and experiencing beautiful things. Before, I forbade myself from doing this because I was given an allowance… So, I said to myself that I have the right to experience things that are beautiful.” (P12, male, 40 yrs).

A quarter of the participants reported an increase in their self-confidence as a result of the TPE sessions. They regained confidence in their ability to manage the fluctuations of their disorder on their own.

“Before, I didn’t even think I could manage it (the disorder). At the slightest problem, I called the psychiatrist, and I realize that now I can fight on my own, not for everything, but there are some things I fight on my own.” (P9, female, 42 yrs).

This increase in self-confidence was accompanied by an increase in assertiveness in everyday life for some participants.

“Since I’ve accepted the fact that I am sick, I also find it less difficult to set my limits, even when I know that others will not necessarily like them. For example, for several months now I’ve been telling myself that I don’t want to go and see my family at Christmas because last year it went very, very badly… I managed to say no to them this year.” (P5, female, 25 yrs).

#### Theme 5: Development of social links

Most of the participants explained that, by the end of the TPE sessions, they felt like they belonged to a group and were finally understood by others. This sense of belonging reduced their feelings of loneliness due to the disorder and consequently their sense of having suffered an injustice.

“I just think that for anyone with or without a disability, being part of a tribe is always a strength. I thought that if I met other people with the same disability who were going through similar things, it would allow me to find strength and in fact to get better and to move forward.” (P16, female, 36 yrs).

As a result of TPE, half of the participants reported that they had expanded their social network, especially by keeping in touch with other members of their group following the end of the TPE cycle. These participants also realized the importance of social connections for maintaining a stable mood and integrating well into society.

“I realized that I shouldn't be locked in with this. I’ve been doing TPE and meditation groups. It only lasts for a while, but it feels good, I feel much less alone, and I’ll probably do discussion groups or something like that again. That’s for sure, that’s a given for me.” (P1, female, 32 yrs).

Some participants also explained that TPE helped them to identify those around them who were genuine supporters and those who were toxic. These participants subsequently decided to maintain contact only with the resourceful people around them.

“To confront myself with reality and with people who live like this. Because in my surroundings, I had so many judgments, criticisms certainly as well, because I did not accept it, but I do not want to hear all that anymore. So, I prefer to see people who really understand and who have the same background.” (P1, female, 32 yrs).

Thus, half of the participants discovered the notion of peer support during the TPE and employed it as a resource following the termination of the sessions.

“We set up a group on WhatsApp; each one of us explains what’s happening to him, and the other one answers by giving him a little help, so there’s a discussion that is created. So, it’s quite nice.” (P13, female, 51 yrs).

#### Theme 6: Change in the management of daily life

Some participants reported a gain in serenity, a general sense of calm in their daily lives following the TPE.

“I feel more at peace with my daily life, whereas this was not the case a few months ago.” (P5, female, 25 yrs).

A few participants explained that they were able to step back from everyday situations and to mitigate their impulsiveness.

“The other evening, I had a fight with a friend… I had a lot of photos of friends hanging on the wall in my room, and I took them all down, and I was close to tearing them all up… but then I said to myself, “wait, you’re not going to do that; you’re going to leave everything on the floor, you’re going to do something else…” and that’s it. Yes, it’s tiring, but I have the impression that there are parts of my daily life where it’s progressing anyway.” (P5, female, 25 yrs).

Finally, a few interviewees noted that their participation in the TPE program helped to increase their freedom of action and that all the benefits of the TPE had opened up new possibilities for them.

“When I was traveling, that was my fear. Because often, when I left to travel, it was actually a problem. But I don’t want to stay at home under the pretext that I have a mental illness, that I’m more fragile and that it could explode. So, to make this crisis plan, to know that I can be in Norway or I don’t know where, to be able to contact my psychiatrist, I can do more things.” (P12, male, 40 yrs).

## Discussion

### General discussion

#### Main findings

Four previous qualitative studies presented the subjective experiences of people with BD following their participation in psychoeducation programs [11–13, 25]. These programs consisted of 8 to 11 sessions of 90 to 120 min, and were led by two facilitators (nurses, psychologists or psychiatrists). Our results, concerning a TPE program, are consistent with these earlier studies: the key elements identified were the establishment of a climate of trust and security, the diversity of the group, and the mutual support that naturally developed among participants. The attitude of facilitators had been highlighted as a key element: Poole et al. noted the importance of a sensitive, flexible and authentic facilitator style [11], and Weiner suggests an attitude of non-judgment and compassion [25]. Patients interviewed by Chen et al. emphasize the benefit of a less didactic teaching posture to facilitate group discussions [12].

The patients of our study reported changes facilitated by TPE: an evolution in their relationship to their troubles, an improvement in their knowledge, understanding and management of their disorders in everyday life, the development of psycho-social skills and social links. Previous studies noted that these programs promoted knowledge through the experiences of others [11, 25], acceptance of disorders [11, 13, 25], and behavioral attitudes toward care [11, 25].

#### Peer-to-peer identification

Sharing experiences with peers is a central component of psycho-educational programs. Peer-to-peer interactions lead participants to identify with the symptoms described by others. It promotes awareness of the disorder, a feeling of being understood by others, a decrease in feelings of loneliness, and a sense of belonging to a group. The notion of perceived social support is highlighted by Poole, and the decrease in the feeling of isolation in all the qualitative studies [11–13, 25]. Group programs have been shown to be superior to individual or internet-based psychoeducation programs8.

These interactions inspire the participants to internally compare the severity of their illness to that of other members of the group. This comparison instils hope for improvement in patients who encounter great difficulties, and it puts the severity of the disorder into perspective for stable patients. The testimony of older people allows young patients to envision themselves in the future despite the disorder. Finally, the diversity of the participants' profiles encourages the participants to compare their life path with those of others. As a result, the positive aspects of each person's life are highlighted, and the efforts made and results achieved are valued. This benefit leads to increases in self-confidence and self-esteem through destigmatization. These observations are very similar to those of Weiner et al., who found the importance of the identification phenomena as well as the differences, concerning the age or the clinical variety of the situations [25]. The mechanisms identified in our study have already been highlighted by other studies, as vectors of both peer-to-peer support and support by peer helpers, even among psychiatric patient populations [26].

#### TPE groups supporting personal recovery process

In this study, the changes described by the TPE participants pertain to each dimension of recovery referenced by the CHIME model [19]. The dimension of connectedness seems to be particularly impacted by TPE, through the development of social connections (i.e., the theme “development of social links”) and incorporates key notions of recovery, such as peer support and the sense of belonging to a group. TPE is also an important source of hope: it instills hope regarding one’s ability to live a fulfilling life once again, ultimately leading to hope for recovery (i.e., the subtheme “hope for recovery”). This hope is nourished by peer helpers, who provide concrete evidence that recovery is possible. As we saw, the identity dimension of CHIME is also relevant to TPE. This identification is mainly positive, as it leads to improved acceptance of bipolar disorder (i.e., the subtheme “better acceptance of the disorder”) and thus to a destigmatization of their condition [11, 25]. The participants feel that they are part of a group whose norms are different from those the general population. Empathy being particularly pronounced in people with BD [27], identification with peers may be particularly pronounced in comparison to other mental disorders. In addition, TPE helps participants reflect on the meaning of their lives. As the disorder is relegated to the background, TPE enables the participants to establish new life projects and to envisage a meaningful and fulfilling life despite their illness (i.e., the subtheme “increased freedom of action”). Finally, by improving their knowledge of the disorder and sharing tools for managing the disorder in everyday life, TPE supports the empowerment of participants. It provides them with the tools they need to make informed decisions regarding their mental health and to take control of their disorder (i.e., the subthemes “acquisition of tools” and “implementation of new strategies”). The patient then feels as if he can communicate actively with caregivers regarding his care (i.e., the subtheme “coconstruction of the treatment with the psychiatrist”) and thus becomes a real actor with respect to his care. Weiner et al. highlighted this feeling of powerful and self-confidence [25].

The main factors facilitating these effects are the sharing of experiences among peers and the provision of knowledge and tools related to the disorder, but our results highlight the importance of the group rules established during the sessions and that of all the elements of posture and facilitation that allow for these secure and constructive discussions.

### Strengths and limitations

Data saturation seemed to be reached, with no new themes emerging from the final interviews. Semistructured interviews featuring open-ended questions minimized the risk of biasing the participants’ discourse. One strength of our study is the fact that the interviewing and coding was carried out blindly by three trained health care professionals who had different profiles; despite this all the themes were identified by the two researchers. Furthermore, the interviews were analyzed inductively, without any preconceived notions regarding the potential results. All these factors ensure the reliability of the results of our study. In addition, the sample used in our study included individuals from different social situations, who were different ages and had very different durations of the progression of the disorder. These participants were also drawn from four separate TPE cycles with different facilitators. The resulting sample improves the reproducibility of our results.

Our study does face some methodological limitations. First, we could have increased the diversity of perspectives in the study by employing other data collection methods or by interviewing the facilitators [28, 29]. In addition, the individual interviews were conducted approximately two months after the end of the TPE sessions. This delay allowed us to objectify the short-term effects of TPE, but it is insufficient to attest to the long-term impact of TPE. Interviews at an even greater remove from the chenconclusion of the TPE would be necessary to evaluate the effects of the TPE over time.

### Clinical implications

National Institute for Health and Care Excellence recommends a combination of pharmacological treatments and structured psychotherapeutic interventions in BD [30].

The lack of data regarding these interventions does not yet allow for more precision in these recommendations. TPE differs from traditional rehabilitative care and therapeutic activities by providing theoretical knowledge concerning bipolar disorder itself. Another unique characteristic of TPE and recovery-oriented care is its horizontal care posture, which allows the experiential knowledge of the participants to be valued as highly as the theoretical input provided by the caregivers; this importance of the facilitator’s attitude to promote recovery is founded in several studies [11, 12, 25]. Unlike other approaches employing peer support, such as discussion groups, TPE offers continuity from one session to the next and allows participants to create deeper connections with one another. However, it would be interesting to study qualitatively and quantitatively the differences in effects between peer support groups and TPE groups, to better understand their respective effects and indications.

The results of our study suggest that TPE could be beneficial for people with bipolar disorder at any point throughout their journey with the disorder. In our study, young people, with a recent diagnosis, reported as many positive effects in their daily life as did people who had been diagnosed with the disorder for more than 20 years. Other quantitative studies have also indicated the benefits of psychoeducation during the early years of bipolar disorder [31, 32].

A recent systematic review by Davenport et al. in 2019 synthesized in a broader way the 10 qualitative studies exploring the experience of bipolar people after different psychotherapeutic care: psychoeducation, cognitive behavioral therapy, mindfulness-based care or interpersonal and social rhythm therapy [33]. Useful elements are highlighted, and are similar to those of our study: i) meeting with peers and sharing experience, ii) an open facilitation style that listens to needs rather than adhering to a rigid structure, enabling communication. Also, the perceived effects seem to overlap with our own results: increased knowledge of BD, mood recognition, control of moods, change of perspective about treatments, responsibility, acceptance and relationships with others. The authors insist on the importance of new outcomes to be considered in evaluative studies, such as empowerment and quality of relationships.

## Conclusions

This qualitative study exploring patients’ perspectives provides evidence to support the benefits of group therapeutic education programs on the personal recovery of people with bipolar disorder. TPE improves all dimensions of recovery, although connectedness, hope and empowerment are the main dimensions impacted. The main effects reported by the participants are an evolution of their relationship with their disorder, an improvement in their knowledge of the disorder, an improvement in their management of the disorder and daily life in general, and their development of psycho-social skills and social relationships. The structure of the program and its facilitation are crucial to ensure these observed effects. To enrich the data concerning this subject and strengthen these results, future research should assess the longer-term effects of TPE on personal recovery and examine the perspectives of caregivers.

## Supplementary Information

**Additional file 1.** Content of the Therapeutic Patient Education program concerning bipolar disorder delivered at the CReSERC.

**Additional file 2.** Semistructured interview schedule.

## Data Availability

The interviews analyzed in this study are available from the corresponding author upon reasonable request. The textual data analyzed in this study, which correspond to the participant interviews, cannot be publicly deposited or transmitted via internet links. We are urged to be cautious by our Hospital Clinical Research Department regarding the transmission of anonymized data. Indeed, in a recent paper published in Nat Commun (PMID: 31,337,762), Rocher and his colleagues stated that “even heavily sampled anonymized datasets were unlikely to satisfy the modern standards for anonymization set forth by GDPR and seriously challenge the technical and legal adequacy of the de-identification release-and-forget model”. Using a generative copula-based method, the authors found that 99.98% of Americans could be correctly reidentified from any dataset using 15 demographic attributes. Moreover, the data collected in this study may include potentially sensitive data (concerning mental health and behavior). Finally, before sending data to other researchers, we must determine that the purpose of the data processing is compatible with the information provided to and consent given by the patients.

## Abbreviations

TPE: Therapeutic Patient Education
CReSERC: Centre Référent en Soins d’Education thérapeutique et de Remédiation Cognitive
MINI: Mini International Neuropsychiatric Interview
MoCA: Montreal Cognitive Assessment
WHO: World Health Organization
