# Supplementary material for: Patients’ perspectives of the effects of a group-based therapeutic patient education program for bipolar disorder: a qualitative analysis
Source: BMC Psychiatry. 2022 Sep 23;22:626. doi: 10.1186/s12888-022-04241-2 (PMC9508709; doi:10.1186/s12888-022-04241-2)
Supplement: Supplementary file 1 — Additional file 1. Content of the Therapeutic Patient Education program concerning bipolar disorder delivered at the CReSERC. [file 12888_2022_4241_MOESM1_ESM.docx]

|  | **Content of the session** |
| --- | --- |
| **Session n°1** | - Introduction to TVE, the programme and the participants - Setting up of group rules - Collective educational diagnosis: representations, knowledge of the disease and treatments, resources, expectations of participants - Self-evaluation of skills |
| **Session n°2** | - The different types of mood disorders - Mania and hypomania: symptoms, prodromes, functional impact, coping strategies |
| **Session n°3** | - Depression: symptoms, prodromes, functional impact, coping strategies - Mixed states: definition, symptoms |
| **Session n°4** | - How to talk about bipolar illness with family and friends - Topics desired by the group: work, parenthood... - Presentation of the next session: knowledge, representations, expectations on the treatment of the disorder |
| **Session n°5** | - Treatments for bipolar disorder: knowledge, representations and expectations about treatments - Neuropshysiology - Medication treatments - Non-drug treatments |
| **Session n°6** | - Living with a bipolar disorder: - vulnerability-stress model - coping strategies: healthy living, stress management - Development of the "crisis plan |
| **Session n°7** | - Preparation of the next session by the participants who wish to do so: choice of themes to be addressed, choice of tools |
| **Session n°8** | - Session with relatives (free session) |
| **Session n°9** | - Summary of the programme and feedback on the session with relatives - Self-evaluation of acquired skills |

**Content of the Therapeutic Patient Education program concerning bipolar disorder delivered at the CReSERC**
