# Supplementary material for: Patients’ perspectives of the effects of a group-based therapeutic patient education program for bipolar disorder: a qualitative analysis
Source: BMC Psychiatry. 2022 Sep 23;22:626. doi: 10.1186/s12888-022-04241-2 (PMC9508709; doi:10.1186/s12888-022-04241-2)
Supplement: Supplementary file 2 — Additional file 2. Semistructured interview schedule. [file 12888_2022_4241_MOESM2_ESM.docx]

**Semi-structured interview schedule**

**Part 1: What supports the well-being of people with bipolar disorder.**

- Can you tell me about what helps you to be well, generally speaking, in your life?

- What has helped you so far in your journey since the first symptoms appeared? What have been your main supports?

- Can you tell me how these things have helped you? How have they been a resource?

- What needs do you think they have met?

**Part 2: Experience and effects of TPE in people with bipolar disorder.**

A) Expectations and motivations for TPE:

- What were your motivations for participating in the TPE sessions?

- What expectations did you have of therapeutic education?

- What was your psychological health before starting the mediation? How did you feel about your life with the disease?

- Had you already participated in this type of mediation in the past?

B) Outcomes of TPE:

- Have you experienced any changes in your life since starting the TPE sessions? Can you describe them to me? Why do you think this has happened?

- Did your participation in the TPE have an impact on your skills, abilities, self-perception?

- Did the effects of the mediation persist after the mediation ended? Can you give me some examples?

- Were there any negative or adverse effects of your participation in the TPE sessions?

C) Evaluation of the TPE process:

- How did you feel during the TPE sessions?

- Which elements of the mediation do you think contributed to the changes that occurred and helped you? Which ones were unpleasant for you?

- More specifically, what did you do during the mediation that helped you move forward?

- What do you think of the relationship that developed during the sessions with the caregivers? Similarly with the other members of the group?

D) Suggestions for improvement:

- Did this type of mediation help you more or less than the mediations you have participated in before? What did she bring you that was different?

- What would you have needed during these sessions to be more helpful?

- What suggestions would you have for improving the TPE sessions so that they are more beneficial to the participants?
